# Association of baseline HBcAg and HBV DNA with persistent detectability of HBV RNA during NA therapy

**DOI:** 10.1371/journal.pone.0352870

**Published:** 2026-07-02

**Authors:** Lanjie Wu, Zixiang Pan, Jianli Yu, Xitan Yue, Fang He, Lili Yu, Ling Pan, Yuyan Wu, Chuntao Zhou, Peiye Yan, Hongying Pan

**Affiliations:** 1 The Second Clinical Medical College, Zhejiang Chinese Medical University, Hangzhou, Zhejiang, China; 2 Department of Infectious Diseases, Zhejiang Provincial People’s Hospital, Affiliated People’s Hospital, Hangzhou Medical College, Hangzhou, Zhejiang, China; 3 Laboratory Medicine Center, Department of Clinical Laboratory, Zhejiang Provincial People’s Hospital, Affiliated People’s Hospital, Hangzhou Medical College, Hangzhou, Zhejiang, China; 4 Cancer Center, Department of Pathology, Zhejiang Provincial People’s Hospital, Affiliated People’s Hospital, Hangzhou Medical College, Hangzhou, Zhejiang, China; 5 Shulan International Medical College, Zhejiang Shuren University, Hangzhou, Zhejiang, China; University of Cincinnati College of Medicine, UNITED STATES OF AMERICA

## Abstract

**Objective:**

Even after long-term nucleos(t)ide analogue (NA) therapy achieves hepatitis B virus (HBV) DNA suppression in patients with chronic hepatitis B (CHB), serum HBV RNA may remain detectable. HBV RNA reflects intrahepatic viral transcriptional activity. This study identified baseline traits and predictors of persistent HBV RNA positivity during NA therapy after sustained HBV DNA suppression.

**Methods:**

This retrospective study included 155 CHB patients who underwent liver biopsy before initiating NA therapy, subsequently achieved sustained HBV DNA suppression on continuous NA therapy. Patients were classified into two groups according to HBV RNA status during therapy. Baseline clinical, virological and histological variables were assessed by multivariate logistic regression. Correlations between HBV RNA and serological markers were also assessed. The diagnostic performance of the model was analyzed using area under the receiver operating characteristic (AUC) and boostrap internal validation.

**Results:**

Overall, 47.7% achieved undetectable serum HBV RNA. Higher baseline HBV DNA and absence of intrahepatic hepatitis B core antigen (HBcAg) independently predicted persistent HBV RNA positivity (both P < 0.001). The combined assessment of HBcAg with HBV DNA improved predictive accuracy (AUC = 0.780), outperforming either marker alone. The AUC value of bootstrap internal validation was 0.756. Across the cohort, HBV RNA showed moderate correlation with hepatitis B surface antigen (HBsAg), hepatitis B e antigen (HBeAg) and hepatitis B e antibody (HBeAb). Correlations were minimal in HBeAg-negative patients and most evident in HBeAg-positive patients.

**Conclusion:**

Incorporating HBcAg histology with baseline HBV DNA may enable more individualized treatment strategies during antiviral therapy. Correlations between HBV RNA and traditional markers are primarily observed in HBeAg-positive patients.

## Introduction

Chronic hepatitis B (CHB) remains a major global health challenge. Hepatitis B virus (HBV) infection affects approximately 296 million people worldwide and causes nearly 1 million deaths annually from HBV-related cirrhosis and hepatocellular carcinoma [1].

Over the past two decades, nucleos(t)ide analogue (NA) have become the cornerstone of CHB therapy by effectively inhibiting HBV replication. It has been reported that more than 90% of patients achieve undetectable HBV DNA after 2–5 years of NA therapy [2–4]. However, most patients require ongoing treatment even with HBV DNA suppression. In theory, a successful cure requires the complete removal of covalently closed circular DNA (cccDNA) [5]. Since NAs are HBV polymerase inhibitors [6], they do not eradicate cccDNA [5]. cccDNA persists at extremely low copy numbers within hepatocyte nuclei and serves as the transcriptional template for all viral RNAs, including pregenomic RNA (pgRNA) and mRNAs encoding HBsAg [5]. During long-term complete virologic suppression with undetectable serum HBV DNA and HBV RNA, circulating HBsAg originates predominantly from integrated HBV DNA, not cccDNA [7]. However, cccDNA retains the potential for viral reactivation. In recent years, several serum markers have been proposed to assess intrahepatic viral activity. Serum HBV RNA has been reported as a potential alternative marker for chronic HBV infection since it provides a direct reflection of cccDNA transcriptional activity [8].

Accumulating clinical evidence highlights the role of serum HBV RNA as a biomarker across patient populations. Studies have linked HBV RNA to treatment endpoints, including hepatitis B e antigen (HBeAg) seroconversion and viral rebound [9–12]. Persistent HBV RNA increases the risk of recurrence after treatment discontinuation, thereby necessitating prolonged therapy and more intensive viral monitoring. Baseline patient characteristics also influence HBV RNA kinetics [13].

Previous studies on HBV RNA kinetics have primarily focused on serum samples due to their ease of collection. However, serology cannot directly reflect viral activity within the liver. HBcAg is a major target of virus-specific T cells. Its expression in hepatocytes leads to altered histological activity, viral replication and immune responses, and it exhibits stronger antigenicity than other viral proteins [14,15]. Although FibroScan has been widely used in clinical practice, it cannot diagnose coexisting intrahepatic pathology and has limited applicability in certain patients [16,17]. Therefore, liver biopsy remains the gold standard.

In this study, we enrolled CHB patients who had achieved HBV DNA < 100 copies/mL for ≥1 year under continuous NA therapy. We evaluated whether combining hepatitis B core antigen (HBcAg) Immunohistochemistry (IHC) with other markers improves prediction of persistent serum HBV RNA positivity. In addition, correlations between HBV RNA and traditional viral markers during treatment were assessed. Baseline features may help identify patients with residual HBV activity despite years of virologic suppression. Such patients may require closer monitoring and adjunctive therapies to suppress the virus. Traditional serology provides limited information in HBeAg-negative patients, making HBV RNA testing a better tool to reveal residual viral activity.

## Materials and methods

### Patients

In this retrospective study, the population comprised 1,365 untreated patients with CHB infection who underwent liver biopsy at Zhejiang Provincial People’s Hospital in Hangzhou, China, between January 2014 and May 2024. Baseline clinical and laboratory data were collected before treatment start.

Inclusion criteria included: positive HBsAg test result for over 6 months; continuous receipt of entecavir (ETV) or tenofovir alafenamide (TAF); completion of serum HBV RNA testing; persistently negative HBV DNA for at least 1 year during NA therapy. Exclusion criteria were as follows: co-infection with other hepatitis viruses (hepatitis A, C, D, or E) or human immunodeficiency virus; the presence of other liver diseases, such as autoimmune hepatitis, alcoholic liver disease, or Wilson disease.

**Fig 1:**
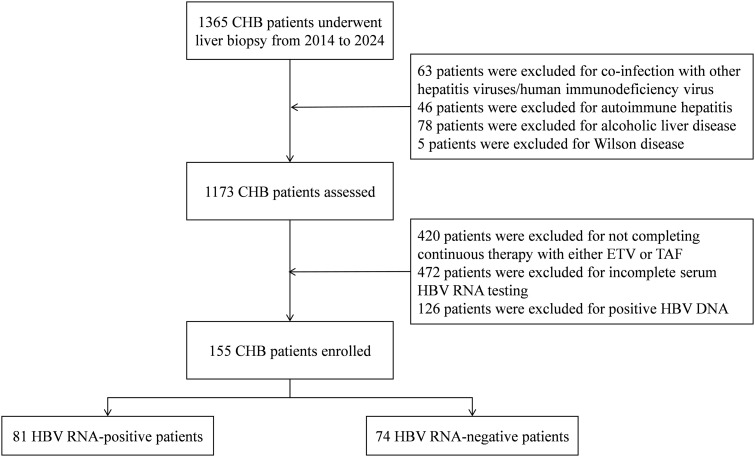
Flow diagram of the study population.

All patients received initial therapy with ETV (0.5 mg qd). Following the availability of TAF, patients were switched to TAF (25 mg qd). Serum HBV RNA and HBV DNA were assessed every 3–6 months. The median follow-up time from treatment initiation to the last visit was 61 months (range, 21–143 months). HBV DNA levels <100 copies/mL were defined as negative HBV DNA, and HBV RNA levels <100 copies/mL were defined as negative HBV RNA. HBV DNA suppression was defined as persistent negative HBV DNA for ≥1 year. Detectable HBV RNA was defined as ≥1 positive result during follow-up after achieving sustained HBV DNA suppression. As shown in Fig 1, our study included 155 patients with CHB. Patients were classified into the HBV RNA-positive group (n = 81) and the HBV RNA-negative group (n = 74) according to HBV RNA status. The study was approved by the Ethics Committee of Zhejiang Provincial People’s Hospital (QT2025171). All research was conducted in accordance with the 1964 Declaration of Helsinki and its later amendments or comparable ethical standards. Since this is a retrospective study, consent was waived by the committee. The data were accessed for research purposes on 10/08/2025.

### Laboratory examinations

The clinical and biochemical parameters were collected at the time of liver biopsy and during treatment, including age, gender, total protein (TP), albumin (ALB), globulin (GLB), alanine aminotransferase (ALT), aspartate aminotransferase (AST), gamma-glutamyltranspeptidase (GGT). The level of HBV DNA, HBV RNA, hepatitis B surface antigen (HBsAg), hepatitis B surface Antibody (HBsAb), HBeAg, hepatitis B e antibody (HBeAb), hepatitis B core antibody (HBcAb) were also collected. HBV RNA quantitative results of <100 copies/mL were replaced with 70.71 copies/mL (100/2^0.5^), whereas results of <50 copies/mL, were replaced with 35.36 copies/mL (50/2^0.5^). The fibrosis-4 index (FIB-4) was calculated as (age × AST) / (platelet count × ALT^0.5^). The aspartate aminotransferase to platelet ratio index (APRI) was calculated as ([AST/ULN] / platelet count) × 100, the gamma glutamyl transpeptidase platelet ratio (GPR) was calculated as (GGT / platelet count) × 100.

### Assessment of liver histology

The liver biopsies were performed blindly under ultrasound guidance with a 16-gauge needle. A 25 mm long biopsy was used to minimize sampling errors. Liver biopsy specimens were immersed in 10% formalin, embedded in paraffin, sectioned and stained with hematoxylin and eosin. Fibrosis stage (0–4) and inflammatory grade (0–4) were assessed according to the Metavir scoring system. Liver histopathology was diagnosed by two experienced pathologists in a double-blind fashion. Intrahepatic HBsAg and HBcAg expression were scored qualitatively by IHC. Significant liver fibrosis was defined as S ≥2, and significant liver inflammation was defined as G ≥ 2.

### Statistical analysis

Statistical analysis were performed using SPSS version 27 and R statistics version 4.5.1. GraphPad Prism version 10.0 was used to peform graphs. Quantitative variables were expressed as mean ± standard deviation or median (interquartile range, 25th–75th percentile) and compared using the Student t test or the nonparametric test (Mann–Whitney). Categorical variables were presented as n (%) and compared using the chi-square (χ²) test. Correlations were assessed using the Spearman rank correlation coefficient.

Logistic regression was used to identify factors associated with detectable HBV RNA among patients who achieved HBV DNA negativity during NA therapy. Receiver operating characteristic curves were applied to evaluate the ability of each parameter to predict persistent HBV RNA positivity. The optimal cut-off value was determined based on the best combination of sensitivity and specificity. Bootstrapping with 1,000 resamples was employed for internal validation.

## Results

### Patient characteristics

Table 1 summarized the baseline clinical and virological characteristics of the patients. Among 155 patients, 47.7% had serum HBV RNA below the lower limit of quantification during NA therapy. Patients were divided into two groups based on their RNA status: RNA-negative and RNA-positive. There were no differences in age, sex, intrahepatic HBsAg expression, G ≥ 2, FIB-4, APRI, GPR, HBsAb, TP, ALB, GLB, ALT, AST, GGT between the two groups (all P > 0.05). However, at baseline, rapid virological response (RVR), intrahepatic HBcAg expression, S ≥ 2, HBeAg, HBeAb, HBcAb, and HBV DNA were associated with RNA status during NA therapy (all P < 0.05). S ≥ 2 (59.3% vs 47.7%, P = 0.03), HBeAg (0.55 [0.35–1264.53] vs 0.37 [0.31–0.56], P < 0.001), HBeAb (0.24 [0.01–45.12] vs 0.02 [0.01–0.91], P = 0.005) and HBV DNA (5.32 [4.11–7.28] vs 3.75 [3.11–5.16], P < 0.001) were significantly higher in the HBV RNA-positive group than in the HBV RNA-negative group. RVR (69.1% vs 85.1%, P = 0.018), intrahepatic HBcAg expression (11.1% vs 24.3%, P = 0.030), and HBcAb (9.07 [8.03–10.49] vs 10.36 [8.77–11.52], P < 0.001) were lower than that of HBV RNA-negative group.

**Table 1:** Baseline clinical characteristics in the HBV RNA-positive and HBV RNA-negative groups.

Characteristic	HBV RNA-positive (n = 81)	HBV RNA-negative (n = 74)	P value
Age, years	44.60 ± 10.18	46.92 ± 10.25	0.161
Male, n (%)	53 (65.4%)	47 (63.5%)	0.803
RVR, n (%)	56 (69.1%)	63 (85.1%)	0.018
Intrahepatic HBsAg expression, n (%)	54 (66.7%)	43 (58.1%)	0.271
Intrahepatic HBcAg expression, n (%)	9 (11.1%)	18 (24.3%)	0.030
Grade ≥2, n (%)	44 (54.3%)	45 (60.8%)	0.414
Stage ≥2, n (%)	48 (59.3%)	56 (47.7%)	0.030
FIB-4	1.16 (0.78, 1.74)	1.09 (0.80, 1.64)	0.694
APRI	0.49 (0.34, 0.77)	0.40 (0.30, 0.67)	0.200
GPR	0.23 (0.15, 0.38)	0.19 (0.12, 0.30)	0.246
HBsAb, mIU/ml	0.38 (0.02, 0.79)	0.50 (0.19, 1.22)	0.059
HBeAg, S/CO	0.55 (0.35, 1264.53)	0.37 (0.31, 0.56)	＜0.001
HBeAb, S/CO	0.24 (0.01, 45.12)	0.02 (0.01, 0.91)	0.005
HBcAb, S/CO	9.07 (8.03, 10.49)	10.36 (8.77, 11.52)	＜0.001
HBV DNA, log10 IU/ml	5.32 (4.11, 7.28)	3.75 (3.11, 5.16)	＜0.001
TP, g/L	72.73 ± 5.35	73.55 ± 4.79	0.316
ALB, g/L	44 (42, 45.55)	44.4 (42.5, 46.53)	0.218
GLB, g/L	29.30 ± 3.98	29.34 ± 3.58	0.954
ALT, U/L	41 (24, 58.5)	35.5 (23, 62.25)	0.744
AST, U/L	34 (25, 43.5)	32.5 (24, 44.25)	0.522
GGT, U/L	23 (16, 35.5)	21.5 (15.75, 33.5)	0.552

Abbreviations: ALB, albumin; ALT, alanine aminotransferase; AST, aspartate transaminase; APRI, aspartate aminotransferase to platelet ratio index; FIB-4, fibrosis-4 index; GLB, globulin; GGT, Gamma Glutamyl transferase; GPR, gamma glutamyl transpeptidase platelet ratio; HBsAg, hepatitis B surface antigen; HBsAb, hepatitis B surface antibody; HBeAg, hepatitis B e antigen; HBeAb, hepatitis B e-antibody; HBcAg, hepatitis B core antigen; HBcAb, hepatitis B core antibody; HBV, hepatitis B virus; RVR, rapid virological response; TP, total protein.

### Baseline predictors of positive HBV RNA

Univariable and multivariable logistic regression analyses were performed to identify baseline factors associated with detectable HBV RNA among patients with sustained HBV DNA suppression on NA therapy. In univariate analysis, RVR (P = 0.021), intrahepatic HBcAg expression (P = 0.034), S ≥ 2 (P = 0.031), baseline HBeAg (P = 0.008), HBeAb (P = 0.015), HBV DNA (P < 0.001) were significantly associated with detectable HBV RNA. Multivariable analysis further showed that the absence of intrahepatic HBcAg expression (P < 0.001) and higher baseline HBV DNA (P < 0.001) were independently associated with detectable HBV RNA (Table 2).

**Table 2:** Logistic regression analysis of factors associated with undetectable HBV RNA.

	Univariate analyses		Multivariate analyses	
	OR (95% CI)	p value	OR (95% CI)	p value
Age	0.978 (0.948-1.009)	0.161		
Gender	0.920 (0.476-1.777)	0.803		
RVR	0.391 (0.177-0.866)	0.021	0.552 (0.190-1.548)	0.262
Intrahepatic HBsAg expression	1.442 (0.750-2.771)	0.272		
Intrahepatic HBcAg expression	2.571 (1.074-6.157)	0.034	8.167 (2.618-30.346)	＜0.001
Grade ≥2	0.766 (0.404-1.453)	0.415		
Stage ≥2	0.468 (0.234-0.934)	0.031	0.567 (0.249-1.273)	0.171
FIB-4	1.076 (0.771-1.503)	0.667		
APRI	1.027 (0.686-1.538)	0.897		
GPR	0.828 (0.492-1.395)	0.478		
HBsAb	0.893 (0.746-1.068)	0.214		
HBeAg	1.001 (1.000-1.001)	0.008		
HBeAb	1.019 (1.004-1.034)	0.015	0.988 (0.962-1.015)	0.357
HBcAb	0.994 (0.952-1.038)	0.791		
HBV DNA	1.471 (1.218-1.777)	＜0.001	1.848 (1.364-2.577)	＜0.001
TP	0.968 (0.909-1.031)	0.315		
ALB	0.953 (0.877-1.035)	0.254		
GLB	0.998 (0.917-1.085)	0.953		
ALT	1.001 (0.997-1.004)	0.752		
AST	1.000 (0.993-1.008)	0.971		
GGT	0.998 (0.991-1.004)	0.485		

Abbreviations: ALB, albumin; ALT, alanine aminotransferase; AST, aspartate transaminase; APRI, aspartate aminotransferase to platelet ratio index; FIB-4, fibrosis-4 index; GLB, globulin; GGT, Gamma Glutamyl transferase; GPR, gamma glutamyl transpeptidase platelet ratio; HBsAg, hepatitis B surface antigen; HBsAb, hepatitis B surface antibody; HBeAg, hepatitis B e antigen; HBeAb, hepatitis B e-antibody; HBcAg, hepatitis B core antigen; HBcAb, hepatitis B core antibody; HBV, hepatitis B virus; RVR, rapid virological response; TP, total protein; OR, odds ratio; CI, confidence interval.

**Fig 2:**
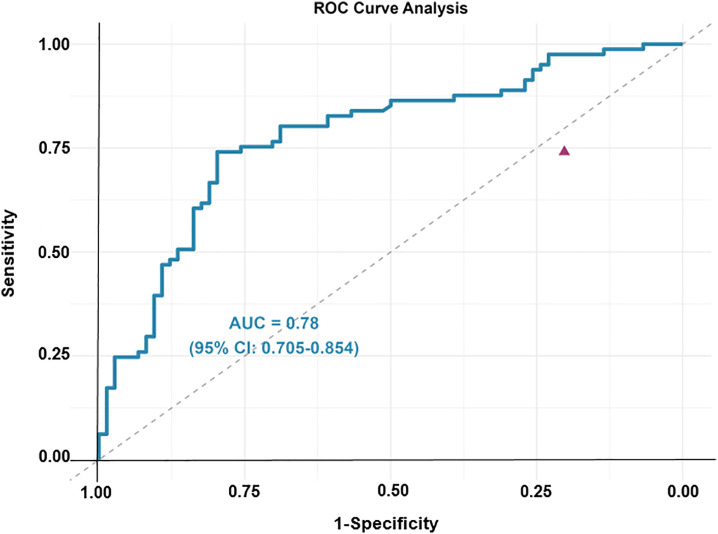
ROC curve of the combination of HBV DNA and HBcAg for predicting persistent HBV RNA positivity.

In order to assess the predictive performance of baseline intrahepatic HBcAg and HBV DNA, area under the receiver operating characteristic (AUC) curves were analysed. The results revealed the combination of HBcAg and HBV DNA (AUC = 0.780 ± 0.074) (Fig 2) had a higher diagnostic value than HBcAg (AUC = 0.566 ± 0.060) or HBV DNA alone (AUC = 0.708 ± 0.082).

ROC analysis further determined an optimal HBV DNA cutoff value of 4.52 log10 IU/mL, yielding a sensitivity of 67.9% and a specificity of 71.6%. However, the AUC of HBV DNA alone was significantly lower than that of the combined model. The cutoff value of combined curve was 0.516, the sensitivity was 74.1% and the specificity was 76.8%. It suggests that patients with baseline risk score <0.516 were more likely to achieve undetectable serum HBV RNA during antiviral therapy.

**Fig 3:**
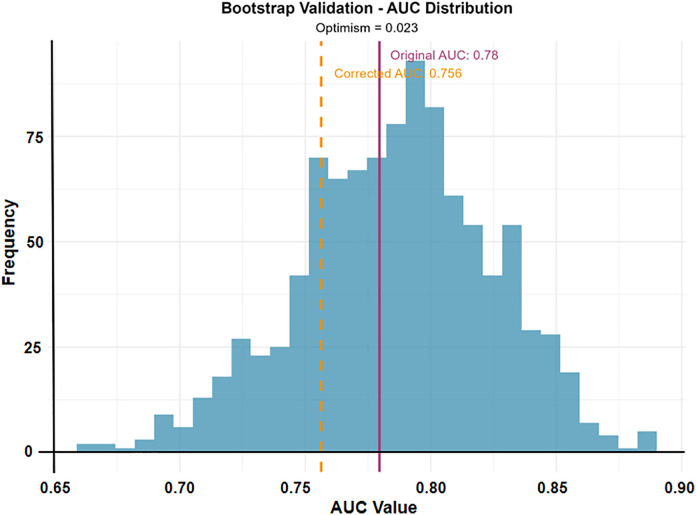
Bootstrap validation of AUC distribution.

The model discrimination analysis yielded an original AUC of 0.780, which decreased to 0.756 after 1,000 bootstrap resamples, with an estimated optimism of 0.023 (Fig 3). These results suggest that the model’s performance is relatively robust and show no significant overfitting.

### Correlations between HBV RNA and viral markers in on-treatment patients

**Fig 4:**
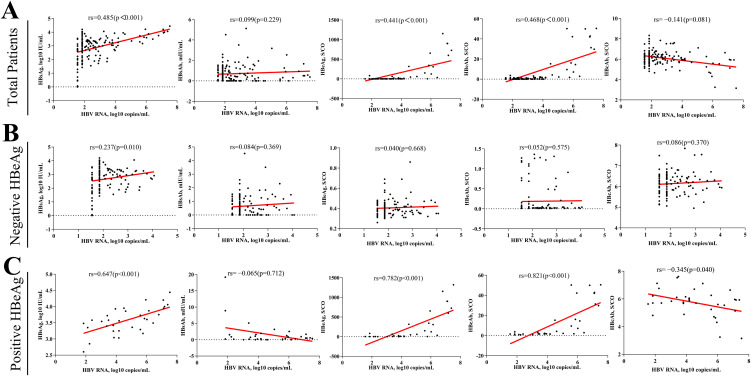
Correlations of on-treatment serum HBV RNA with HBsAg, HBsAb, HBeAg, HBeAb, HBcAb. (A) Total patients; (B) HBeAg-negative patients; (C) HBeAg-positive patients.

To further investigate the value of serum HBV RNA for monitoring patients receiving NA therapy with suppressed HBV DNA, Spearman correlation analysis was performed between HBV RNA and virological markers (Fig 4). Overall, HBV RNA was moderately correlated with on-treatment HBsAg (rs = 0.485, P < 0.001), HBeAg (rs = 0.441, P < 0.001), and HBeAb (rs = 0.468, P < 0.001) but was not correlated with HBsAb (rs = 0.099, P = 0.229) or HBcAb (rs = −0.141, P = 0.081).

In HBeAg-negative patients, HBV RNA correlated only with HBsAg (rs = 0.237, P = 0.010) and not with HBsAb (rs = 0.084, P = 0.369), HBeAg (rs = 0.040, P = 0.668), HBeAb (rs = 0.052, P = 0.575), or HBcAb (rs = 0.086, P = 0.370).

In HBeAg-positive patients, HBV RNA correlated strongly with HBeAg (rs = 0.782, P < 0.001) and HBeAb (rs = 0.821, P < 0.001), and moderately with HBsAg (rs = 0.647, P < 0.001). HBV RNA also showed a weak, inverse correlation with HBcAb (rs = −0.345, P = 0.040). No correlation was observed between HBV RNA and HBsAb (rs = −0.065, P = 0.712).

## Discussion

Although NAs effectively suppress HBV replication, they do not directly affect intranuclear cccDNA, leading to persistent viral transcription. Studies have shown that serum HBV RNA is positively correlated with intrahepatic cccDNA [11,18], making it a potential biomarker for assessing viral activity. In this cohort of 155 CHB patients receiving long-term NA therapy, though serum HBV DNA was effectively suppressed, 52.3% had detectable serum HBV RNA. This indicates that even after effective suppression of serum HBV DNA, HBV RNA continues to reflect the transcriptional activity of cccDNA. Patients who test positive for HBV RNA should not discontinue treatment, as this indicates that true transcriptional control has not been achieved.

We investigated the ability of serum virological markers alone or in combination with liver histology for HBV RNA outcomes during NA therapy. Additionally, correlations between HBV RNA and serological markers were assessed in HBeAg-positive and HBeAg-negative subgroups. High baseline HBV DNA and absent HBcAg expression were associated with HBV RNA positivity.

High baseline HBV DNA was closely associated with the persistent HBV RNA. This finding supports the view of Liu et al.[19]. They proposed that NAs inhibit HBV by blocking reverse transcription of pgRNA into DNA, while the viral RNA transcription and translation machinery remain intact. Accordingly, patients initiating therapy with high viral loads may have a larger intrahepatic cccDNA reservoir, leading to prolonged persistence of serum RNA during therapy. Choi et al. also reported that baseline HBV DNA remained significantly associated with poor outcomes after long-term treatment [20]. The above evidence suggest that patients with high baseline HBV DNA may require prolonged therapy or additional interventions.

In our study, the absence of intrahepatic HBcAg expression was associated with detectable HBV RNA. The chronic progression of HBV infection may be due to inadequate cellular immune function. Mechanistically, HBcAg elicits an immune response primarily through its role in triggering adaptive immunity, specifically by stimulating CD8 + T cells [21]. When HBV infects hepatocytes, HBcAg is presented on the surface of infected cells, which are recognized by HBV-specific CD8 + T cells. These T cells then produce antiviral cytokines and engage in cytotoxic activity to eliminate the infected hepatocytes. The absence of HBcAg expression may permit viral persistence by evading immune clearance [22]. Although absent HBcAg expression before NA therapy may indicate low viral activity, it also implies that the immune system may be insufficient to achieve complete viral clearance. For patients with absent hepatic HBcAg expression prior to treatment, combination therapy of immunotherapies with NA may facilitate viral clearance.

Overall, serum HBV RNA was moderately correlated with HBsAg (rs = 0.485, P ＜ 0.001). This suggests that higher RNA levels are associated with greater HBsAg production. In our study, a clear correlation between HBV RNA and HBsAg was observed in treated HBeAg-positive patients, indicating ongoing cccDNA transcriptional activity in those without seroconversion. The correlation was weaker in HBeAg-negative patients, consistent with the findings of Thompson et al.[23]. Mechanistically, in HBeAg-negative CHB, HBsAg largely derives from integrated HBV genomic sequences rather than cccDNA [7]. Therefore, the overall correlation between HBsAg and HBV RNA is largely driven by HBeAg-positive patients; after seroconversion, the correlation essentially disappears. HBV RNA appears more reliable than HBsAg for monitoring residual viral transcriptional activity [24]. Given the weak association observed in HBeAg-negative patients, our findings suggest that HBV RNA reflects transcripyional activity not captured by HBsAg. If the predictive value of high baseline HBV DNA and absence of intrahepatic HBcAg for persistent HBV RNA positivity is confirmed in independent prospective studies, HBV RNA or these baseline predictors could serve as an adjunctive treatment endpoint in HBeAg-negative CHB.

In HBeAg-positive patients, serum HBV RNA correlated strongly with HBeAg levels (rs = 0.782, P < 0.001). An entecavir treated cohort study found that HBeAg levels could predict HBV RNA in HBeAg-positive CHB, and the correlation strengthened as treatment continued[25]. These observations suggest that both serum HBV RNA and HBeAg may serve as surrogates of nuclear cccDNA transcriptional activity. A strong correlation was observed between HBV RNA and HBeAb in HBeAg-positive patients (rs = 0.821, P < 0.001). This seems against common sense, given that HBeAb typically emerges after HBeAg clearance. However, during HBeAg seroconversion, HBeAg and HBeAb can coexist temporarily. One interpretation is that patients without HBeAb had the highest HBV RNA levels, whereas early appearance of HBeAb was accompanied by a decline in RNA levels. In cross-sectional analysis, this pattern may present as a direct correlation.

As expected, HBV RNA showed no significant correlation with HBsAb or HBcAb. In our chronically infected cohort, HBsAb was largely undetectable, accounting for the absence of any association. Interestingly, HBcAb showed no correlation with HBV RNA.It consistents with prior studies [25,26]: although HBcAb levels can mark the immune phase, it does not directly reflect viral replication in patients. In HBeAg-positive subgroups, HBV RNA and HBcAb exhibited a weak inverse correlation. Therefore, HBcAb is not a practical indicator of residual HBV transcription during receiving antiviral therapy. In summary, HBsAb and HBcAb primarily reflect host immune activity rather than real-time viral activity. Accordingly, they lack correlation with HBV RNA.

In HBeAg-negative patients, HBV RNA showed no significant correlation with HBsAb, HBeAg, HBeAb, or HBcAb. This finding is consistent with a recent study [27].

However, there are some limitations in our study. First, this was a single-center retrospective analysis with 155 patients, which limits generalizability. Second, HBV genotype, cccDNA levels and intrahepatic RNA were not measured, preventing mechanistic insights into why high baseline HBV DNA and absent intrahepatic HBcAg might predict fluctuations in serum HBV RNA. Third, HBcAg status was determined from a single liver biopsy, which is susceptible to sampling error. Fourth, the single observation of HBV RNA exceeding 100 copies/mL may represent a transient elevation rather than sustained viral transcriptional activity.

## Conclusion

In conclusion, despite successful suppression of HBV DNA during NA therapy, persistent serum HBV RNA positivity remains common. Our study emphasizes that higher baseline HBV DNA and absence of intrahepatic HBcAg are important independent predictors of persistent HBV RNA. These predictive factors help provide individualized decision-making when considering whether to use NA combination therapy or immunotherapies. For patients with suppressed HBV DNA, monitoring serum HBV RNA may better reflect viral activity. Future studies should focus on the mechanisms behind HBV RNA persistence, particularly in the absence of detectable HBV DNA, and exploring its potential as a biomarker for assessing treatment efficacy and risk of relapse.
